# Acute Podocyte Vascular Endothelial Growth Factor (VEGF-A) Knockdown Disrupts alpha_V_beta_3_ Integrin Signaling in the Glomerulus

**DOI:** 10.1371/journal.pone.0040589

**Published:** 2012-07-13

**Authors:** Delma Veron, Guillermo Villegas, Pardeep Kumar Aggarwal, Claudia Bertuccio, Juan Jimenez, Heino Velazquez, Kimberly Reidy, Dale R. Abrahamson, Gilbert Moeckel, Michael Kashgarian, Alda Tufro

**Affiliations:** 1 Department of Pediatrics, Yale University School of Medicine, New Haven, Connecticut, United States of America; 2 Department of Pediatrics, Albert Einstein College of Medicine, Bronx, New York, United States of America; 3 Analytical Imaging Facility, Albert Einstein College of Medicine, Bronx, New York, United States of America; 4 Department of Internal Medicine, Yale University School of Medicine, New Haven, Connecticut, United States of America; 5 Department of Anatomy and Cell Biology, University of Kansas Medical Center, Kansas City, Kansas, United States of America; 6 Department of Pathology, Yale University School of Medicine, New Haven, Connecticut, United States of America; Institut National de la Santé et de la Recherche Médicale, France

## Abstract

Podocyte or endothelial cell VEGF-A knockout causes thrombotic microangiopathy in adult mice. To study the mechanism involved in acute and local injury caused by low podocyte VEGF-A we developed an inducible, podocyte-specific VEGF-A knockdown mouse, and we generated an immortalized podocyte cell line (VEGF^KD^) that downregulates VEGF-A upon doxycycline exposure. *Tet-O-siVEGF:podocin-rtTA* mice express *VEGF* shRNA in podocytes in a doxycycline-regulated manner, decreasing VEGF-A mRNA and VEGF-A protein levels in isolated glomeruli to ∼20% of non-induced controls and urine VEGF-A to ∼30% of control values a week after doxycycline induction. Induced *tet-O-siVEGF:podocin-rtTA* mice developed acute renal failure and proteinuria, associated with mesangiolysis and microaneurisms. Glomerular ultrastructure revealed endothelial cell swelling, GBM lamination and podocyte effacement. VEGF knockdown decreased podocyte fibronectin and glomerular endothelial alpha_V_beta_3_ integrin *in vivo*. VEGF receptor-2 (VEGFR2) interacts with beta_3_ integrin and neuropilin-1 in the kidney *in vivo* and in VEGF^KD^ podocytes. Podocyte VEGF knockdown disrupts alpha_V_beta_3_ integrin activation in glomeruli, detected by WOW1-Fab. VEGF silencing in cultured VEGF^KD^ podocytes downregulates fibronectin and disrupts alpha_V_beta_3_ integrin activation cell-autonomously. Collectively, these studies indicate that podocyte VEGF-A regulates alpha_V_beta_3_ integrin signaling in the glomerulus, and that podocyte VEGF knockdown disrupts alpha_V_beta_3_ integrin activity via decreased VEGFR2 signaling, thereby damaging the three layers of the glomerular filtration barrier, causing proteinuria and acute renal failure.

## Introduction

Vascular endothelial grown factor-A (VEGF-A) is essential for angiogenesis, endothelial cell proliferation, migration, and survival [1], [2]. The biological activity of VEGF-A is mediated mainly by VEGF receptor 2 (VEGFR2) signaling, which is influenced by neuropilin-1, a co-receptor for several VEGF isoforms [3], [4], while VEGF receptor 1 (VEGFR1) functions as a decoy [5]. VEGF-A is required to maintain the glomerular filtration barrier structure, and hence for normal renal function. Genetic deletion of VEGF-A in the endothelium leads to systemic endothelial degeneration, vascular thrombosis and swelling of glomerular endothelium [6]. Podocyte VEGF-A deletion and excess soluble VEGFR1 (sFlt-1, a soluble VEGF receptor that acts as a ligand trap) cause thrombotic microangiopathy and hypertension [7]–[9]. In humans, decreased VEGF-A availability due to preeclampsia or cancer treatment with VEGF/VEGFR2 antagonists is associated with proteinuria, hypertension and thrombotic microangiopathy [9].

VEGF-A function at the glomerular filtration barrier is tightly regulated in a dose and age dependent manner. Moderate podocyte VEGF overexpression induces different renal disorders during development and in adult life [10], [11]. We reported congenital nephrotic syndrome and minimal change disease associated with moderate podocyte VEGF_164_ overexpression at different stages of mouse kidney development [10]. By contrast, podocyte VEGF_164_ overexpression in adult mice induced glomerular lesions indistinguishable from early diabetic glomerulopathy [11]. Deletion of podocyte VEGF-A in developing mice prevented glomerular vascularization and glomerular filtration barrier development [12]. In adult mice, chronic VEGF-A knockout induced thrombotic microangiopathy [9]. The acute effects of podocyte VEGF downregulation and the molecular mechanism whereby the lack of VEGF damages the glomerular filtration barrier are unclear.

Angiogenic factors, integrins and extracellular matrix proteins work in concert in the angiogenic process [13], [14]. Integrins are adhesion receptors that link the extracellular matrix to the cell cytoskeleton. Integrins are composed of two subunits, a large alpha chain and a smaller beta chain. Extracellular matrix proteins laminin, collagen, and fibronectin bind beta1 integrin subunit [15]. Integrins are ubiquitously localized in the kidney [16]. Integrin alpha3beta1 is essential for podocyte development and function, and for assembly of the GBM [17], [18]. Deletion of alpha3 integrin caused kidney and lung abnormalities, specifically, decreased branching of glomerular capillaries, disrupted glomerular basement membrane (GBM) organization and podocyte foot process differentiation, causing proteinuria and perinatal lethality [19]. Deletion of alpha3 integrin limited to podocytes resulted in massive proteinuria and GBM lamination [20]. Podocyte beta_1_ integrin deletion caused proteinuria at birth, associated with podocyte loss, capillary and mesangial degeneration leading to end-stage renal failure [21]. Even though beta1 integrin expression by podocytes is required to maintain glomerular structural integrity, other integrins are also important. Integrin alpha_V_beta_3_ is expressed in endothelium, mesangial cells and podocytes from rodents and humans [22], [23]. Beta_3_ integrin-deficient mice, a model of Glanzmann thrombasthenia, die due to hemorrhage, with vascularized kidneys and glomerular lesions [24]. Integrin alpha_V_ null mice are embryonic or perinatal lethal, but no overt vascular or renal defect has been reported [25].

Activation of alpha_V_beta_3_ integrin by soluble urokinase receptor (suPAR) has been shown to cause proteinuria and focal segmental glomerulosclerosis in mice and humans [26]–[27]. In endothelial cells, vascular smooth muscle cells and fibroblasts, alpha_V_beta_3_ integrin interacts with VEGFR2, and this hetero-receptor complex is important during physiological and pathological angiogenesis [27]–[29].

To study the molecular mechanism involved in the pathogenic effects of podocyte VEGF knockdown, we developed a mouse model that silences all VEGF isoforms using an inducible shRNA approach, and we generated an immortalized podocyte cell line that downregulates VEGF-A upon doxycycline exposure. Here, we analyzed the effect of acute podocyte VEGF knockdown in mice, and show it causes acute renal failure and proteinuria. We also show that VEGF knockdown induces decreased VEGFR2 autocrine and paracrine signaling, fibronectin and alpha_V_beta_3_ integrin downregulation, disrupting VEGFR2-alpha_V_beta_3_ integrin functional interaction, thereby damaging the three layers of the glomerular filtration barrier.

## Results

### Doxycycline-induced Podocyte VEGF-A Knockdown Mediated by shRNA in Mice

**Figure 1:**
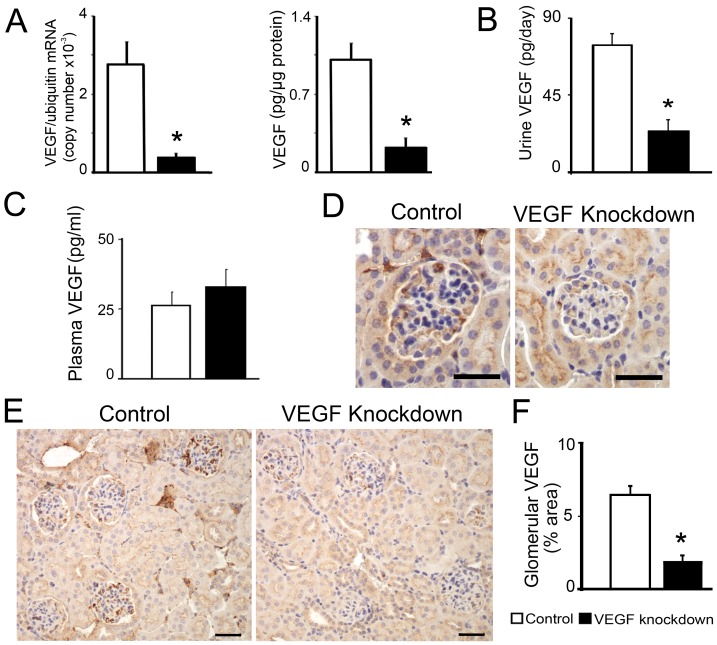
VEGF knockdown mouse model. (A) One week after doxycycline-induction, *podocin-rtTA:tet-O-siVEGF* adult mice decrease VEGF-A mRNA and VEGF-A protein levels to ∼20% of controls in isolated glomeruli. (B) Urine VEGF-A decreases in VEGF knockdown mice to ∼30% of control values. (C) Plasma VEGF-A is similar in VEGF knockdown and control mice. (D) VEGF immunohistochemistry shows normal expression in control (left), and decreased glomerular VEGF-A expression in VEGF knockdown mice (right), scale bar = 30 µm. (E) Low magnification VEGF immunohistochemistry shows absence of VEGF in some glomeruli in VEGF knockdown mice (right), (scale bar = 40 µm). (F) Glomerular VEGF-A (VEGF^+^ area/glomerular area x100) in VEGF knockdown mice (n = 135) decreases to ∼30% of controls (n = 118). In all bar graphs * indicates P<0.05 compared to control.

We generated a transgenic mouse carrying a podocin promoter-driven shRNA targeting the first exon of mouse VEGF-A under the control of the tetracycline reverse transcriptional activator *(podocin-rtTA:tet-O-siVEGF)*. Induction of podocyte *VEGF* shRNA expression by doxycycline for one week in *podocin-rtTA:tet-O-siVEGF* (*siVEGF*) adult mice decreased VEGF-A mRNA and VEGF-A protein levels in isolated glomeruli to ∼20% of induced single transgenic or non-induced *siVEGF* controls, determined by qPCR and ELISA, respectively (Figure 1A). Urine VEGF-A decreased in VEGF knockdown mice to ∼30% of control values (Figure 1B), whereas circulating VEGF-A was similar to control mice (Figure 1C). VEGF-A immunohistochemistry also showed diminished VEGF-A in glomeruli from VEGF knockdown mice compared to controls (Figure 1D–F), including multiple glomeruli lacking immunoreative VEGF (48/135 vs. 13/118, VEGF knockdown vs. controls, p<0.05). Body weight, kidney weight, hematocrit and urine volume were similar in control and VEGF knockdown mice (Table 1). Together these data suggest that *VEGF* shRNA expression silenced podocyte VEGF-A in vivo, decreasing local VEGF-A in glomeruli and urine without altering systemic VEGF-A.

**Table 1:** General parameters from *siVEGF* mice.

	Control	VEGF knockdown
**Body weight (g)**	28.8±1.3	26.1±1
**Kidney weight (mg)**	218.2±10	206.4±10
**Hematocrit (%)**	45.2±0.6	45.4±0.8
**Urine ml/day**	0.28±0.04	0.24±0.01

### Doxycycline-induced Podocyte VEGF-A Knockdown in Cultured Podocytes

**Figure 2:**
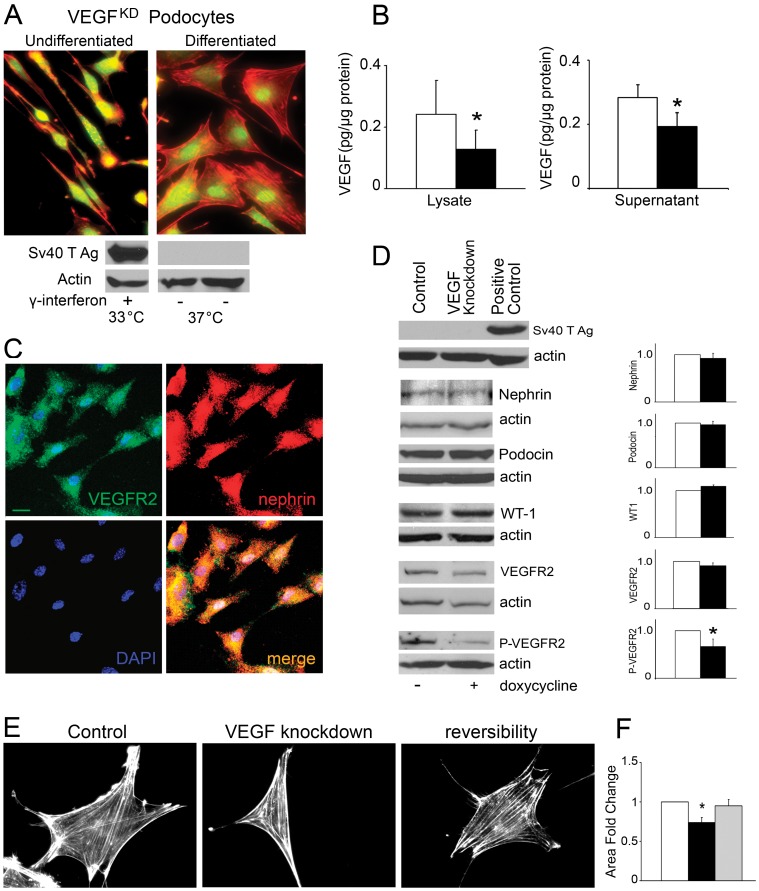
VEGF knockdown podocyte model. (A) Cell Tracker (green) and rhodamine phalloidin (red) labeling shows images of spindle-like undifferentiated VEGF^KD^ podocytes (top left) and rhomboidal/polygonal differentiated VEGF^KD^ podocytes (top right). Immunoblots show Sv40T antigen expressed only in undifferentiated VEGF^KD^ podocytes (bottom left). (B) Differentiated VEGF^KD^ podocytes exposed to doxycycline decreased VEGF-A cellular content and secreted VEGF-A. (C) Immunocytochemistry: differentiated VEGF^KD^ podocytes in control conditions express VEGFR2 (green) and nephrin (red), which co-localize (yellow, merge), cell nuclei labeled with DAPI (blue); scale bar = 10 µm. (D) Immunoblots show that VEGF-A knockdown did not change nephrin, podocin, WT-1 and VEGFR2 expression level in VEGF^KD^ podocytes; whereas VEGF-A knockdown decreased ^Y1175^VEGFR2 phosphorylation as compared to control. Bar graphs show densitometric analysis of ≥3 immnublots/protein expressed as fold change mean ±SEM. (E) VEGF-A knockdown changed VEGF^KD^ podocyte shape and decreased their size, assessed by rhodamine phalloidin staining, which were reversible upon exposure to recombinant VEGF_165_. (F) Quantitation of VEGF^KD^ podocyte area change induced by VEGF knockdown, reversibility by exposure to VEGF_165_, expressed as fold change mean±SEM. Scale bar = 20 µm. In all bar graphs * indicate P<0.05 vs. control.

A cloned immortalized podocyte cell line was derived from *siVEGF* mice harboring doxycycline-regulated VEGF knockdown (VEGF^KD^). VEGF^KD^ podocytes proliferated and expressed SV40 T antigen in permissive conditions consistent with undifferentiated podocytes (Figure 2A). After a week on non-permissive conditions, cell shape changed from cobblestone to arborized podocytes, and SV40 T antigen was no longer expressed (Figure 2A). Exposure of differentiated podocytes to doxycycline for 48 hours resulted in ∼50% decrease of VEGF-A cell content, and ∼30% decrease in secreted VEGF-A, as compared to control (Figure 2B). VEGFR2 and nephrin co-localized within podocytes (Figure 2C), as previously described [30]. Nephrin, podocin, WT1 and VEGFR2 protein levels in differentiated podocytes were not altered by VEGF downregulation (Figure 2D). By contrast, VEGF knockdown caused a significant decrease in ^Y1175^VEGFR2 phosphorylation (Figure 2D). In addition, VEGF knockdown induced changes in podocyte shape and size, decreasing cell surface area significantly, which were rescued by addition of recombinant VEGF_165_ (Figure 2E–F), suggesting that decreased VEGFR2 signaling may impair podocyte adhesion or modulate podocyte cytoskeleton.

### Podocyte VEGF Knockdown Damaged the Glomerular Filtration Barrier Leading to Proteinuria and Renal Failure

**Figure 3:**
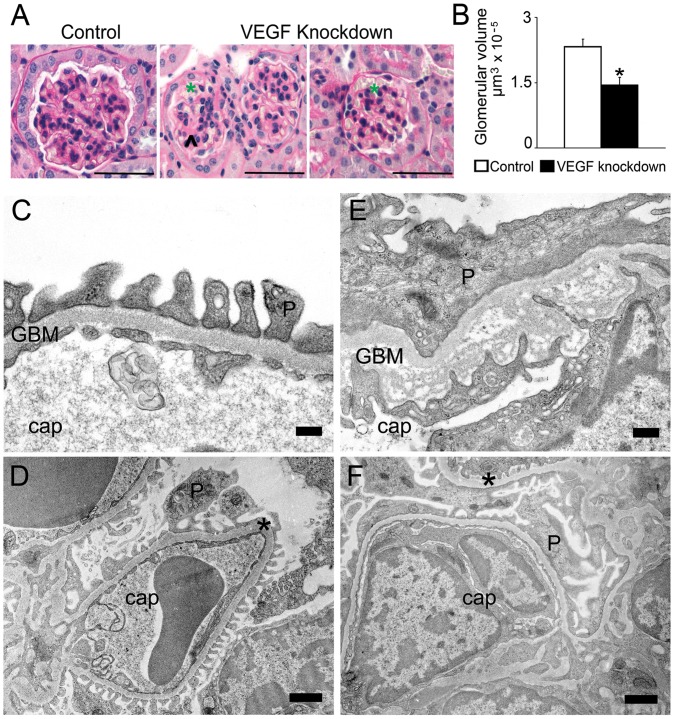
VEGF knockdown glomerular phenotype. (A) PAS stain shows mesangiolysis (arrowhead), microaneurisms (green asterisk) and decreased glomerular volume in VEGF-A knockdown glomeruli, scale bars  = 50 µm. (B) Quantitation of glomerular volume reveals that VEGF knockdown induces significant decrease in glomerular size. * indicates P<0.05 vs. control. (C-D) TEM: control glomeruli show normal ultrastructure; scale bars  = 200 nm (C) and 1 µm (D). (E-F) TEM: VEGF knockdown glomeruli show endothelial cell swelling, vacuolization and decreased fenestration; GBM lamination of the lamina densa, irregular thickening and interdigitations of the endothelium; podocyte foot process effacement; scale bars = 500′nm (E) and 1 µm (F). Cap = capillary, P = podocyte, *  = GBM.

Light microscopy examination showed that, after one week of doxycycline induction, short term VEGF knockdown in podocytes decreases glomerular volume ∼30% compared to controls, and induces mesangiolysis and glomerular microaneurisms (Figure 3A–B). No thrombi were identified. To determine whether the decreased glomerular size was due to apoptosis we performed TUNEL assay; no changes in the number of apoptotic cells/glomerulus were detected: 1/163 glomeruli vs. 0/175 glomeruli, VEGF knockdown (n = 5) vs. control (n = 4), p = 0.37.

The glomerular filtration barrier is composed of fenestrated endothelium, glomerular basement membrane and podocytes. Control mice showed normal glomerular filtration barrier ultrastructure (Figure 3C–D). VEGF knockdown caused ultrastructural damage of the whole glomerular filtration barrier (Figure 3E–F): swelling, vacuolization and decreased fenestration of endothelial cells; glomerular basement membrane lamination, expansion of the lamina densa, irregular thickening and interdigitations of the endothelium surface; and podocyte foot process effacement.

**Figure 4:**
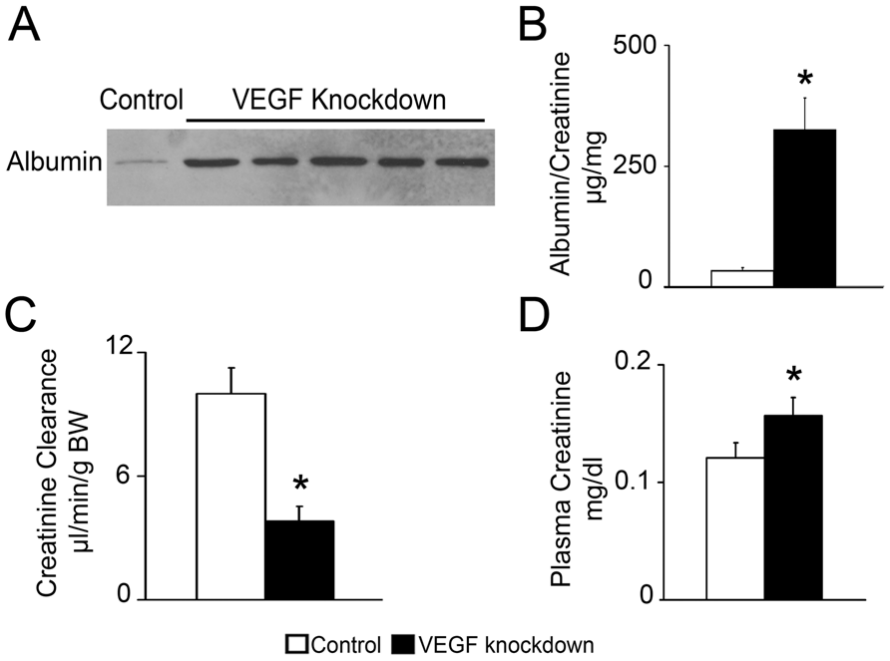
VEGF knockdown induces proteinuria and acute renal failure. (A) Immunoblot shows severe albuminuria in VEGF Knockdown mice. (B) ELISA: urinary albumin/creatinine ratio in VEGF knockdown mice is 10-fold higher than in controls. (C) Creatinine clearance is 62% lower in VEGF knockdown mice than in controls. (D) Plasma creatinine significantly increases in VEGF knockdown mice. * indicates P<0.05 vs. control.

Acute functional abnormalities were observed in VEGF knockdown mice. Severe proteinuria was demonstrated by urinary albumin immunoblot and albumin/creatinine ratio 10-fold higher than controls (Figure 4A–B). VEGF knockdown mice had glomerular filtration rate 62% lower than controls, measured by creatinine clearance (Figure 4C), and significantly increased plasma creatinine (Figure 4D). Blood pressure, measured by telemetry, was normal before induction and during VEGF knockdown (Table 2). No significant changes in systolic, diastolic blood pressure or heart rate were observed throughout the study (Table 2 and Figure S1). Together, these data suggest that podocyte VEGF knockdown caused acute renal failure by damaging all glomerular filtration barrier components, with a distinct lamination of the lamina densa and extensive endothelial damage in addition to podocyte effacement, in the absence of hypertension.

**Table 2:** Blood Pressure in *siVEGF* mice.

	Control	VEGF knockdown
**Systolic blood pressure (mmHg)**		
Peak	136.1±4.8	141.18±7.3
Rest	115.56±5.3	116.7±3.9
**Diastolic blood pressure (mmHg)**		
Peak	92.7±4	97.0±7.1
Rest	78.8±2.9	82.1±4

Systolic blood pressure and diastolic blood pressure is normal in VEGF knockdown mice. In control period and VEGF knockdown period, values are mean ±SE in peak of activity (dark period) and rest (light period).

### Podocyte VEGF Knockdown Decreases Fibronectin Expression

**Figure 5:**
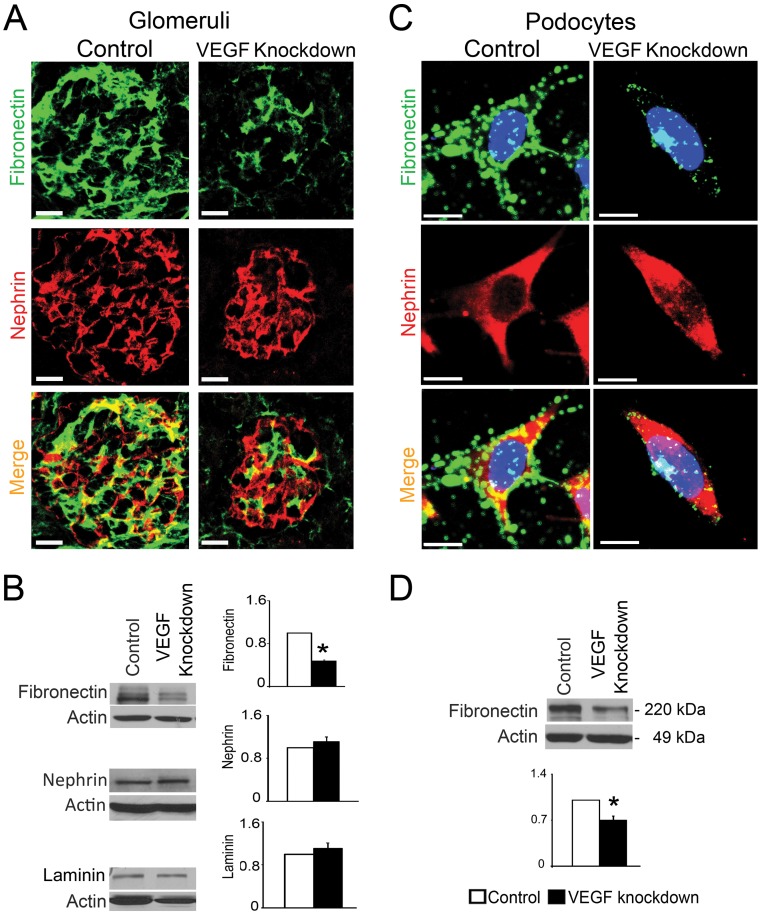
VEGF knockdown downregulates fibronectin expression. (A) IHC: VEGF knockdown glomeruli show marked decrease in immunoreactive fibronectin, while nephrin expression is unchanged. Scale bar = 20 µm (B) Representative immunoblots show decreased fibronectin in VEGF knockdown kidney lysate, whereas nephrin and laminin expression levels are similar to controls. (C) ICC: Fibronectin expression decreases in doxycycline-induced VEGF^KD^ podocytes, while nephrin does not change. (D) Representative immunoblot shows fibronectin downregulation in induced VEGF^KD^ podocytes, scale bars = 10 µm. In (C) and (D) bar graphs show densitometric analysis, data are expressed as mean±SEM fold change in arbitrary units as compared to controls, n≥3, * indicate P<0.05 vs. control.

To gain insight into the observed abnormalities of the lamina densa we examined the protein expression of the main components of the GBM. Total laminin protein level and localization were similar in control and VEGF knockdown mice (Figure 5B, Figure S2). Total collagen IV and alpha1-alpha5 collagen IV were normally localized in control and VEGF knockdown mice (Figure S2 and data not shown). By contrast, fibronectin was significantly decreased in kidney lysates from VEGF knockdown mice as compared to controls (Figure 5B). Fibronectin co-localization with nephrin decreased in VEGF knockdown glomeruli (Figure 5A, Figure S3), suggesting that fibronectin was reduced in podocytes, while nephrin expression did not change by immunoblot or immunohistochemistry (Figure 5A–B). To further evaluate these in vivo findings, we examined fibronectin expression in VEGF^KD^ cultured podocytes. Notably, podocyte VEGF knockdown significantly decreased fibronectin expression in cultured podocytes, as assessed by immunocytochemistry and immunoblot (Figure 5C–D). These data indicate that podocyte VEGF knockdown cell autonomously downregulates fibronectin expression.

### VEGF Knockdown Decreases Endothelial alpha_V_beta_3_ Integrin

**Figure 6:**
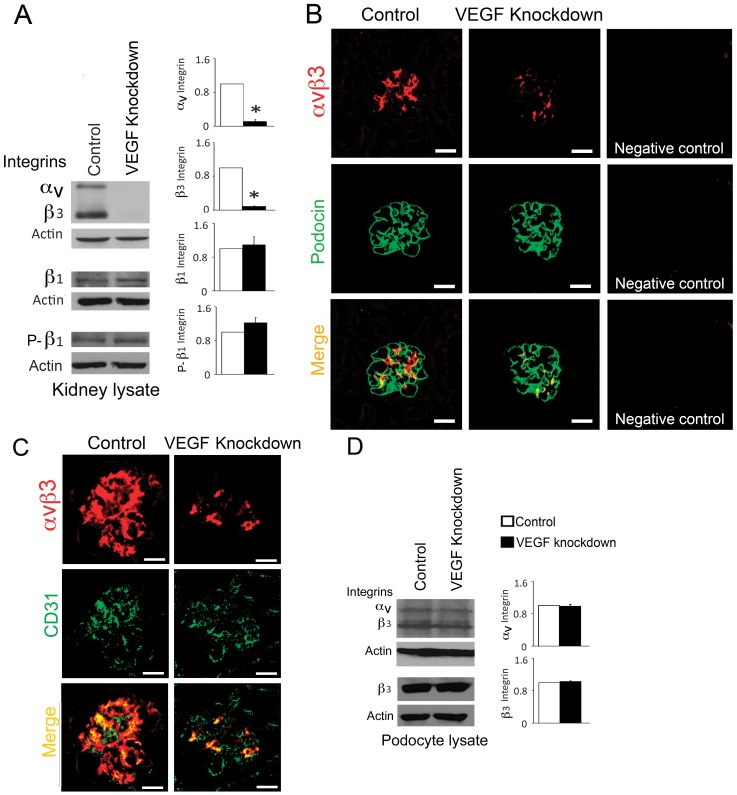
Podocyte VEGF knockdown downregulates endothelial alpha_V_beta3 integrin. (A) Representative immunoblots show decreased alpha_V_beta_3_ integrin in VEGF knockdown kidney lysate, while beta_1_ integrin and S^785^- beta_1_ integrin (P-β1) remain at control levels. (B) Dual-immunostaining shows decreased alpha_V_beta_3_ integrin in VEGF knockdown glomeruli, with minimal co-localization with podocin, which is stable; negative controls shown. (C) Dual-immunostaining shows alpha_V_beta_3_ integrin and CD31 (endothelial marker) co-localization in control glomeruli, while in VEGF knockdown alpha_V_beta_3_ integrin decreases and CD31 does not. Note that immunoreactive alpha_V_beta_3_ signals appear higher than in (B) due to permeabilization required to detect CD31. (D) Representative immunoblots show alpha_V_beta_3_ integrin and beta_3_ integrin levels in podocyte lysate unchanged upon VEGF knockdown. In (A) and (D) bar graphs show densitometric analysis, data are expressed as mean±SEM fold change as compared to controls, n≥3, * indicate P<0.05 vs. control. In (B) and (C) scale bars = 20 µm.

Integrin alpha_V_beta_3_ plays an important role in angiogenesis and in hypertension-induced vascular remodeling [31]. Even though alpha_V_beta_3_ integrin is the primary vitronectin receptor, it also binds fibronectin [31]. Podocyte VEGF knockdown in mice induced significant downregulation of alpha_V_beta_3_ integrin, while beta_1_ integrin level and beta_1_ integrin S^785^ phosphorylation were not altered in kidney lysates (Figure 6A). Moreover, podocyte VEGF knockdown decreased alpha_V_beta_3_ integrin in the glomerular endothelium, as shown by dual immunostaining, where alpha_V_beta_3_ co-localized mostly with the endothelial marker CD31, and marginally with podocin (Figure 6B–C and Figure S4). In cultured podocytes alpha_V_beta_3_ integrin expression level was not altered by VEGF knockdown (Figure 6D). These findings suggest that *in vivo* podocyte VEGF knockdown decreases alpha_V_beta_3_ integrin non-cell autonomously in glomerular endothelial cells.

### VEGFR2 and alpha_V_beta_3_ Integrin Interact in Podocytes

**Figure 7:**
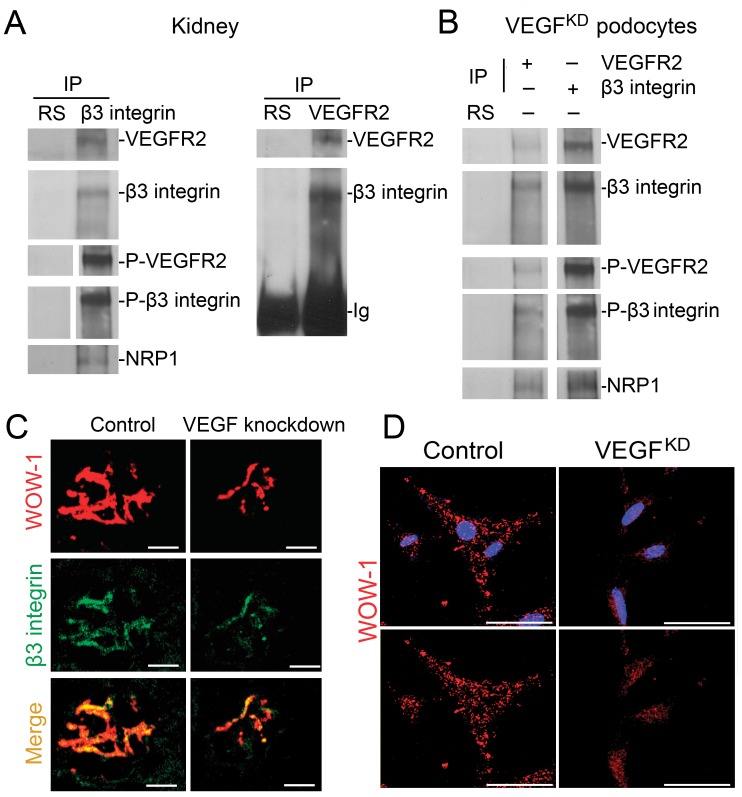
VEGFR2-β3 integrin-neuropilin-1 interact *in vivo* and in cultured podocytes. VEGF knockdown decreases alpha_V_beta_3_ integrin activity. (A) VEGFR2 - beta_3_ integrin - neuropilin1 (NRP1) co-immunoprecipitate *in vivo,* shown by reciprocal VEGFR2 and beta_3_ integrin IP. Negative control is rabbit serum (RS). Immunoprecipitates were blotted with VEGFR2, beta_3_ integrin, Y^1175^-VEGFR2, anti S^785^- beta_3_ integrin and neuropilin-1 antibodies. (B) VEGFR2 - beta_3_ integrin – NRP1 co-immunoprecipitate in cultured podocytes. IPs were performed as described in (A) using VEGF^KD^ podocyte lysates. (C) Dual-immunostaining shows decreased active alpha_v_beta_3_ integrin (WOW-1) and total beta_3_ integrin in glomeruli from VEGF knockdown mice. (D) Immunocytochemistry shows decreased active alpha_V_beta_3_ integrin (WOW-1) in VEGF knockdown as compared to control podocytes, blue nuclei (Hoechst 33342). Scale bars = 20 µm.

The relationship between alpha_V_beta_3_ integrin and VEGFR2 is crucial in the endothelium for physiological and pathological angiogenesis [29], [32], [33]. We examined this relationship in kidney lysates and VEGF^KD^ cultured podocytes. VEGFR2 and beta_3_ integrin co-immunoprecipitate *in vivo* and in VEGF^KD^ cultured podocytes (Figure 7A–B). Moreover, neuropilin-1 also participates in this multi-protein complex (Figure 7A–B). Podocyte VEGF knockdown decreases inside-out alpha_V_beta_3_ integrin activation in vivo (Figure 7C), as assessed by immunolabeling with WOW1-Fab, which detects active alpha_V_beta_3_ integrin exclusively [34], suggesting that VEGF signaling regulates the activity of the integrin receptor complex *in vivo*. In cultured podocytes, VEGFR2, beta_3_ integrin and neuropilin-1 interact (Figure 7B), and VEGF knockdown decreases beta_3_ integrin activity (Figure 7D) without altering alpha_V_beta_3_ integrin expression levels (Figure 6D). Outside-in alpha_V_beta_3_ integrin activation, assessed by AP5 immunostaining was not altered by VEGF knockdown *in vivo* or VEGF^KD^ podocytes (Figure S5). Together, these findings suggest that VEGF-A signals modulate podocyte alpha_V_beta_3_ integrin activity cell autonomously, by modifying VEGFR2- alpha_V_beta_3_ integrin crosstalk.

## Discussion

VEGF-A is essential for the development and maintenance of the glomerular filtration barrier [12]. Our studies uncover a specific molecular mechanism mediating VEGF-A requirement in the adult glomerulus. We show that *in vivo* podocyte VEGF-A regulates fibronectin and alpha_V_beta_3_ integrin signaling in the glomerulus, and that disruption of VEGFR2- alpha_V_beta_3_ integrin crosstalk by acute podocyte VEGF knockdown damages the three layers of the glomerular filtration barrier, resulting in proteinuria and acute renal failure.

We generated a transgenic mouse model to knockdown VEGF in podocytes by doxycycline-regulated shRNA, enabling to study the pathogenic effects of local VEGF downregulation and repair processes *in vivo* in a reversible manner, at difference from permanent loss of VEGF expression in knockout models. An immortalized podocyte cell line derived from these mice silences VEGF-A upon doxycycline exposure, decreasing both VEGF cell content and VEGF secretion. Differentiated VEGF^KD^ podocytes express prototypical podocyte proteins: nephrin, podocin, WT1, as well as VEGFR2 [35], [36], [30]. Accordingly, doxycycline-induced VEGF knockdown decreased VEGFR2 phosphorylation in VEGF^KD^ podocytes, resulting in cell shape and size change. Lee et al. reported a similar autocrine response in endothelial cells [6].

A surprising finding of this study was that *in vivo* podocyte VEGF knockdown for only one week induced proteinuria and kidney failure (Fig. 4). Even though the circulating VEGF levels were normal (Fig. 1), short-term podocyte VEGF knockdown caused a glomerular phenotype characterized by low glomerular volume, mesangiolysis, microaneurisms and typical features of endotheliosis, including endothelial cell swelling, interdigitation into the GBM, widening and lamination of the lamina densa, and podocyte effacement. These abnormalities were not due to hypertension, microangiopathic anemia or decreased availability of circulating VEGF. Instead, they resulted from decreased VEGFR2 signaling in the glomerulus. The evidence for this conclusion is that mice with VEGF knockdown were normotensive, their hematocrit and VEGF circulating levels were normal, while VEGF mRNA and protein were significantly decreased in isolated glomeruli, and in the urine (Figure 1). Accordingly, podocyte VEGF knockdown decreased VEGFR2 phosphorylation in cultured podocytes (Figure 2). We previously reported that absence of VEGF-A signals promotes VEGFR2-nephrin interaction [30]. This may have contributed to maintain normal nephrin expression in the setting of proteinuria. Alternatively, the latter could be due to the short duration of the experiment.

Podocyte VEGF knockdown glomerular phenotype is reminiscent of preeclampsia, an entity due, at least in part, to excess soluble VEGFR1 acting as a decoy receptor, effectively decreasing circulating VEGF availability [7], [8]. A previous report showed that long-term deletion of VEGF in podocytes, using a different genetic approach, induces thrombotic microangiopathy and hypertension [9]. Our short-term podocyte VEGF knockdown did not develop such a severe phenotype, as determined by light microscopy, TEM examination and normal blood pressure. Instead, the glomerular phenotype induced by short-term podocyte VEGF knockdown was similar to milder cases of renal disease induced by anti-VEGF drugs 37,38, supporting the concept that disruption of the tight regulation of glomerular VEGF causes acute and chronic renal disease.

An important conclusion drawn from our experiments is that decreased autocrine and paracrine VEGFR2 signaling induced by podocyte VEGF knockdown disrupts VEGFR2- alpha_V_beta_3_ integrin crosstalk at the glomerular filtration barrier. The experimental evidence supporting this is as follows. First, podocyte VEGF knockdown downregulates alpha_V_beta_3_ integrin in glomerular endothelial cells (Figure 6). Second, VEGFR2 interacts with beta_3_ integrin and neuropilin-1 in the kidney *in vivo* and in cultured podocytes (Figure 7). Third, podocyte VEGF knockdown disrupts alpha_V_beta_3_ integrin activation in glomeruli (Figure 7). Fourth, in cultured podocytes alpha_V_beta_3_ integrin activation is negatively modulated cell-autonomously by VEGF knockdown, leading to reversible changes in podocyte shape and size (Figures 2 and 7).

In endothelial cells VEGF-A signaling activates integrin alpha_V_beta_3_ via VEGFR2 [39]–[40]. VEGF-induced alpha_V_beta_3_ integrin activation is dependent on affinity modulation and VEGFR2-beta_3_ integrin direct association [39]. This inside-out mechanism of alpha_V_beta_3_ integrin activation is consistent with our observation of decreased WOW1 and baseline AP5 labeling in VEGF knockdown glomeruli and podocytes. The interaction of VEGFR2- alpha_V_beta_3_ integrin is a complex critical modulator of angiogenesis in vitro and in vivo [40]–[42]. In cultured endothelial cells beta_3_ integrin silencing impairs cell adhesion, migration and capillary growth in response to VEGF [43]. However, beta_3_ integrin null mice have increased VEGFR2 signaling [42]. Beta_3_ integrin negatively regulates VEGF-mediated angiogenesis by limiting neuropilin-1 interaction with VEGFR2 [40]. Thus, we propose that low alpha_V_beta_3_ integrin activity plays a compensatory role in the setting of decreased glomerular VEGFR2 signaling.

VEGF-A stimulates uPAR expression in endothelial cells [44]. Excessive soluble uPAR, a biomarker of systemic inflammation [45], was shown to cause FSGS in mice and humans by binding and activating podocyte beta_3_ integrin [26], [27]. Whether VEGF-A signaling regulates suPAR effects at the glomerular filtration barrier, remains to be determined.

Podocyte VEGF knockdown-induced disruption of VEGFR2 - alpha_V_beta_3_ integrin signaling at the glomerular filtration barrier resulted in endothelial injury and GBM lamination. VEGF and alpha_V_beta_3_ integrin normally provide endothelial cell survival signals, stimulate adhesion and fenestrae formation [43]. Endotheliosis-associated GBM lamination appears to be due to defective alpha_V_beta_3_ integrin-mediated endothelial adhesion or altered assembly of the GBM due to lower expression of fibronectin. Integrin alpha_3_beta_1_ is crucial for podocyte development and GBM assembly [17], [18]. Deletion of alpha_3_ integrin caused disorganized GBM, associated with proteinuria and perinatal lethality [19]. Podocyte-specific alpha_3_ integrin deletion resulted in massive proteinuria and GBM lamination [20]. Podocyte beta_1_ integrin deletion resulted in effaced podocytes, multilaminated GBM, expansion of the lamina rara externa, and normal glomerular endothelium [18]–[21]. Although podocyte VEGF knockdown induced GBM lamination in adult mice, the GBM splitting expanded the lamina rara interna adjacent to damaged endothelial cells. Consistent with this, podocyte VEGF knockdown did not alter beta_1_ integrin expression or phosphorylation.

In summary, our studies showed that acute podocyte VEGF knockdown in mice decreases autocrine and paracrine VEGFR2 signaling, induces fibronectin and alpha_V_beta_3_ integrin downregulation and decreased activation in the glomerulus. Further, podocyte VEGF knockdown disrupted VEGFR2- alpha_V_beta_3_ integrin functional interaction in the glomerulus, thereby damaging the three layers of the glomerular filtration barrier, resulting in proteinuria and acute renal failure. Collectively, our findings provide mechanistic insight on potential targets for intervention in pathological circumstances where kidney VEGF is disregulated.

## Materials and Methods

### Generation of Inducible, Podocyte-specific VEGF-A Silencing in Mice

A shRNA targeting the first exon of mouse VEGF (Acc# M95200.1) was selected using siRNA Designer algorithm (Clontech). Oligonucleotides (Operon) consisted of a Bam H1 overhang on the 5′ end of the duplex; 19 nucleotides of the shRNA sense strand (top strand: 5′-ccatgaagtgatcaagttc-3′); a loop sequence (top strand: 5′-ttcaagagagaacttgatcacttcatgg-3′); a Pol III termination site of 6 consecutive thymidine residues; a Mlu site to verify cloned inserts; and an EcoRI overhang on the 3′end of the duplex. The double stranded DNA was cloned between the Bam H1 and EcoR1 site of a self inactivating retroviral expression vector (RNAi-Ready-pSiren-RetroQ-TetH, Clontech) [46], that expresses a ds short hairpin RNA under the control of the modified Tet-responsive promoter derived from the P_Tremod_ and the human U6 promoters. Functionality of the construct was assayed by transfection on Hela Tet-On cells (Clontech #630901) and induction with doxycycline [1 µg/ml] for 48 hs. Cells were lysed and VEGF expression analyzed by western blot (Fig. S6). The *tet-O-siVEGF* construct was purified by electrophoresis and DNA extraction (QIAEXII gel extraction kit, (Qiagen). The purified construct DNA was introduced into fertilized oocytes from FVB mice by pronuclear injection using standard techniques. Transgenic *tet-O-siVEGF* mice were identified by PCR using the following primers: 5′-CGTATGTCGAGGTAGGCGTGT-3′ and 5′-TGCTGTCCATCTGCACGAG-3′). Transgenic *tet-O-siVEGF* were crossbred with *podocin-rtTA* mice, kindly provided by J. Kopp (NIH), and genotyped as described [47]. Double transgenic mice *tet-O-siVEGF:podocin-rtTA* are viable, healthy and fertile. All mouse protocols were approved by the AECOM and Yale Committees for Animal Use and Experimentation.

Adult *tet-O-siVEGF:podocin-rtTA* mice, 12±0.6 weeks of age were induced with doxycycline (0.625 mg/g chow, Harlen Teklar) during 1 week (VEGF knockdown, n = 26), or fed standard diet (Control, n = 19). Additional controls were used, single transgenic mice (ST + dox, *tet-O-siVEGF* or *podocin-rtTA*) fed doxycycline chow for 1 week (n = 9), for VEGF quantification by mRNA and ELISA to rule out ‘leakage’ and for phenotype characterization (light and electron microscopy, proteinuria). At the end of the study period a 24 hours urine collection was obtained in metabolic cages, blood was obtained by venous puncture, kidneys were harvested and mice were euthanized under anesthesia. Glomeruli were isolated as described [48], RNA and protein were isolated from glomeruli. Creatinine was measured in plasma and urine by HPLC [49], and clearance was calculated. Albuminuria was measured by ELISA (Albuwell-M-Elisa, Exocell), in 24 hour samples and expressed as albumin:creatinine ratio (µg/mg).

### Telemetry Blood Pressure Measurement

A radiotelemetric blood pressure transducer was placed into the carotid artery of *tet-O-siVEGF:podocin-rtTA* mice as described [50], mice were allowed a week to recover. Recovery was deemed appropriate when blood pressure recordings were stable and had normal diurnal variation for three consecutive days. Mice were singly housed, placed on a special receiver unit that monitors all parameters every 5 min using DataQuest System (Data Sciences, St. Paul, MN), and had free access to water and chow. During 12 h light (rest) and dark (activity) cycles, systolic and diastolic blood pressure, pulse pressure, heart rate, and activity level were recoded and averaged over 6 h periods [50]. After blood pressure recordings were stable, a 3-day baseline was obtained for the 5 parameters (control) on standard diet, followed by seven days on doxycycline containing chow (VEGF knockdown).

### Generation of Podocyte Cell Line with Inducible VEGF Silencing

To generate a conditionally immortalized podocyte cell line with doxycycline-inducible VEGF knockdown, we crossbred *tet-O-siVEGF:podocin-rtTA* mice with *H-2Kb-tsA58* mice (Immortomouse®, Jackson Laboratory, Bar Harbor, ME). Glomeruli were isolated from triple transgenic mice [48], cultured on collagen-I coated plates in RPMI1640 medium with 10% FBS, 100 U/ml penicillin/streptomycin, nystatin 50 U/ml, 100 U/ml mouse γ interferon at 33°C, in air/5% CO_2_ (permissive conditions) [35]. Podocytes were expanded in permissive conditions, and cloned by dilution cloning. Clones were selected according to morphology, podocyte-specific protein expression and response to doxycycline, expanded on collagen I under permissive conditions with 10 U/ml γ interferon. Podocyte VEGF^KD^ differentiation was induced by γ interferon removal from culture medium and temperature shift to 37°C (non-permissive conditions) for ≥7 days in order to inactivate the SV40 T antigen. Differentiated VEGF^KD^ podocyte at 70–80% of confluence were incubated in RPMI 1640 medium, 10% tet-system FBS (Clontech), 100 U/ml penicillin/streptomycin, 50 U/ml nystatin and 50 U/ml heparin (Control), or added 1 µg/ml doxycycline (VEGF knockdown) for 2 days. Podocytes were serum starved for 8 hours before all experiments. Cells were harvested in lysis buffer (1% TritonX-100, 1% Na DOC, 0.1%SDS, 20 mMTris, 0.16 M NaCl, 1 mM EDTA, 15 mM NaF, 1 mM EGTA), 1 mM Na_2_VO_4_ and protease inhibitor cocktail (Roche) were added. Total protein and VEGF-A were measured in cell lysate and supernatant, using BCA (BioRad) and ELISA (mVEGF, R&D), respectively, following the manufacturers’ instructions.

### Histology, Morphometric Analysis and Transmission Electron Microscopy (TEM)

Kidneys were fixed in 10% formalin, embedded in paraffin and processed for light microscopy. Hematoxylin-eosin and PAS staining were performed to evaluate histological changes. Glomerular volume was determined in 4 mice per group, as previously reported [11], [51]. Glomerular diameters were measured in 104.1±10 glomeruli/section at X400 magnification. Kidney cortex was fixed with 2% paraformaldehyde and 2.5% glutaraldehyde in 0.1 M sodium cacodylate buffer and processed for TEM and viewed on a JEOL 1200EX, as previously described [11], [51].

### Immunoblotting

Pooled samples of whole kidney lysates were generated using equal amount of protein from each mouse (n = 5 per group), 80–200 µg protein were resolved by 8–10% SDS/PAGE and immunoblotted, as previously described [11]. The following primary antibodies were used: anti-WT-1 (Santa Cruz, sc192); anti-nephrin (Fitzgerald, 20R-NP002); anti-podocin (Sigma PO372,); anti-laminin (Sigma L9393); anti fibronectin (Sigma F3648), anti alpha_V_beta_3_ integrin (Millipore MAB 1976); anti beta_3_ integrin (Cell Signaling 4702); anti-p Tyr^747^ beta_3_ integrin (Santa Cruz sc-101707); anti beta_1_ integrin (Millipore 44–870G); anti beta_1_ pS^785^ integrin (Invitrogen 9271); anti pTyr^1175^-VEGFR2 (Cell signaling); anti-actin (Sigma, A2066) and anti-BSA (Upstate, 07–248). Anti-rabbit and anti-mouse (Jackson ImmunoResearch Laboratories), and anti-guinea pig (Fitzgerald) horseradish peroxidase-conjugated (HRP) antibodies were used as secondary antibodies, and visualized by enhanced chemiluminescence (ECL, Amersham Biosciences). Densitometric analysis was performed using ImageJ (NIH) software, and data were expressed as fold change from control samples.

### Immunoprecipitation

VEGF^KD^ podocytes exposed to medium with or without doxycycline (1 µg/ml) for 48 hours, were lysed in immunoprecipitation buffer (1% Triton X-100, 1% Nonidet P-40, 0.5% Na deoxycholate, 150 mM NaCl, 10 mM Tris pH 7.5, 1 mM EDTA, 50 mM NaF) and protease inhibitor mixture (Roche Diagnostics); lysates (1 mg) were cleared by centrifugation. Pooled whole kidney lysates (1.5 mg) were resuspended in IP buffer. Lysates were pre-cleared with protein A-agarose beads (Roche), incubated with either VEGFR2 (sc-504, Santa Cruz) or beta_3_-integrin (sc-14009, Santa Cruz) antibody overnight at 4°C, followed by incubation with protein A-agarose (Roche) for 4 h. Rabbit serum was used as negative control. Agarose beads were washed 4 times with IP buffer and bound proteins were eluted in Laemmli’s sample buffer (Sigma). Immunoprecipitates were analyzed by SDS-PAGE and western blotting using VEGFR2 (#2479), integrin beta_3_ (#4702), and Tyr^1175^VEGFR2 (#2478) from Cell Signaling, S^785^-integrin beta_3_ (sc-101707, Santa Cruz) and neuropilin-1 (gift from A. Kolodkin) primary antibodies, incubated with HRP-conjugated anti-rabbit secondary antibody (GE Healthcare) and detected by chemiluminescence. Lysates from HEK cells transfected with VEGFR2 or whole kidney were used as positive controls. Each immunoprecipitation experiment was performed at least 3 times.

### Immunohistochemistry and Immunocytochemistry

Kidneys were incubated in 18% sucrose, embedded in Tissue-Tek Cryo-OCT Compound (Fisher Scientific) and frozen in isopentane/dry ice or formalin-fixed and paraffin embedded. Cryosections (10 µm) were fixed in −20°C acetone for 10 min. Cryosections were permeabilized with 0.3% Triton X, blocked with 5% donkey serum in PBS or TBS buffer and incubated with nephrin (Fitzgerald, 20R-NP002), fibronectin (Sigma F3648), alpha_V_beta_3_ integrin (Millipore, MAB 1976), podocin (Sigma PO372, 1∶1000), CD31 (BD Pharmingen™) diluted in PBS-Tween+BSA, beta_3_ integrin (Santa Cruz, SC 14009), WOW-1 (gift from S. Shattil), AP5 (GPIIIa, clone AP5, Gen-Probe GTI-N7P) or laminin (Sigma L9393) antibodies. Paraffin sections were deparaffinized, incubated in 10 mM citrate, blocked and incubated with anti-VEGF antibody (Dako, M7273), or anti collagen IV (Southern Biotech). Appropriate fluorescent-tagged Cy2 or Cy3-labelled secondary antibodies, and Hoechst 33342, nucleus marker (Invitrogen) were used. VEGF-A immunohistochemistry was performed as previously described [11]. To quantitate VEGF knockdown the glomerular area with immunoreactive VEGF and the corresponding total glomerular area were measured using Image J (NIH: http://rsbweb.nih.gov/ij/docs/examples/stained-sections/index.html) in control and VEGF knockdown glomeruli from 3–4 mice/group.

Podocytes labeled with Cell Tracker® (Invitrogen) following the manufacturer’s instructions, were fixed with 4% paraformaldehyde and incubated with rhodamine phalloidin (Invitrogen) 60 min at room temperature to label F-actin. Differentiated VEGF^KD^ podocytes (1.2×10^5^) plated on collagen I-coated glass slide chambers, kept in standard medium or doxycycline for 48 hours, were fixed in 4% paraformaldehyde and stained with rhodamin phalloidin or blocked with 5% donkey solution and incubated with primary antibodies for immunocytochemistry as described above. For reversibility experiments, VEGF^KD^ podocytes were exposed to doxycycline for 48 hours, then 50 ng/ml of recombinant VEGF_165_ (R&D) was added to the culture medium for 8 hours. Images were obtained by confocal microscopy (Olympus FluoView300). Podocyte surface area was measured using Image J (NIH: http://rsbweb.nih.gov/ij/docs/examples/stained-sections/index.html) in control (n = 77), VEGF^KD^ (n = 84), and reversibility conditions (n = 89) from four independent experiments.

### Real-time PCR

Total RNA was isolated from isolated glomeruli from induced (+dox) and uninduced (-dox) *tet-O-siVEGF:podocin-rtTA* mice as previously described^11^. Reverse transcription products were combined into two separate pools (+dox and -dox). Real-time PCR amplifications were performed in triplicate as previously described [11]. Experiments were repeated at least three times. Data were normalized to ubiquitin and expressed as copy number ×10^−3^, VEGF primers used were previously described [11]. Ubiquitin primers were: 5′-CCCATCACACCCAAGAACAAG-3′ and 5′-TGCGAGTTCCGTCTGCTGT-3′.

### Statistical Analysis

All values are expressed as mean ± SEM. To determine statistical significance, we used unpaired Student t-test, paired Student t-test for comparisons of blood pressure values before and after doxycycline treatment, and ANOVA followed by Bonferroni correction for analysis of podocyte area changes and VEGF-A immunostaining. P<0.05 was deemed statistically significant.

## Supporting Information

Figure S1 **Podocyte VEGF Knockdown mice have normal blood pressure.** Graph represents the average over 6 h periods from systolic blood pressure, diastolic blood pressure, pulse pressure and heart rate recorded every 5 min. All parameters show similar patterns during control period (standard diet) and VEGF knockdown period (doxycycline diet), n = 4 mice.
(TIF)

Figure S2 **Podocyte VEGF knockdown does not alter total laminin or total collagenIV in the kidney.** IHC: laminin (green) and collagenIV (red) low magnification images show similar localization pattern in control and VEGF knockdown kidneys. Scale bars  = 100 µm.
(TIF)

Figure S3 **Podocyte VEGF knockdown downregulates glomerular fibronectin.** IHC: fibronectin (green) and nephrin (red) low magnification images show decreased fibronectin and preserved nephrin in VEGF knockdown glomeruli. Decreased merged signals (yellow) suggest that fibronectin is decreased in podocytes. Scale bars  = 100 µm.
(TIF)

Figure S4 **VEGF-A knockdown decreases glomerular alpha_v_ beta_3_ activity.** Dual-immunostaining shows decreased active alpha_v_beta_3_ integrin (WOW-1) and total beta_3_ integrin in glomeruli from VEGF knockdown mice, and demonstrates that alpha_v_beta_3_ integrin expression is limited to glomeruli; scale bars = 100 µm.
(TIF)

Figure S5 **Podocyte VEGF knockdown does not alter outside-in alpha_v_ beta_3_ activation.** IHC: AP5 immunolabeling is similar in control and VEGF knockdown glomeruli (A), and podocytes, even after exposure to VEGF_165_ (B), suggesting that VEGF knockdown does not modulate outside-in alpha_v_ beta_3_ activation. Scale bars  = 50 µm.
(TIF)

Figure S6 **VEGF-A knockdown by shRNA in culture.** *VEGF* shRNA construct was transfected into HeLa-tet-on cells, clones 1 and 5 were induced with 1 µg/ml doxycycline; proteins were extracted after 48 hours and analyzed by western blotting using a polyclonal anti-VEGF antibody (SC#507). Control cells transfected with empty vector are compared with induced clones 1 and 5 showing ∼90% inhibition of protein expression.
(TIF)
