# Supplementary figures and images for: Acute Podocyte Vascular Endothelial Growth Factor (VEGF-A) Knockdown Disrupts alphaVbeta3 Integrin Signaling in the Glomerulus
Source: PLoS One. 2012 Jul 13;7(7):e40589. doi: 10.1371/journal.pone.0040589 (PMC3396653; doi:10.1371/journal.pone.0040589)

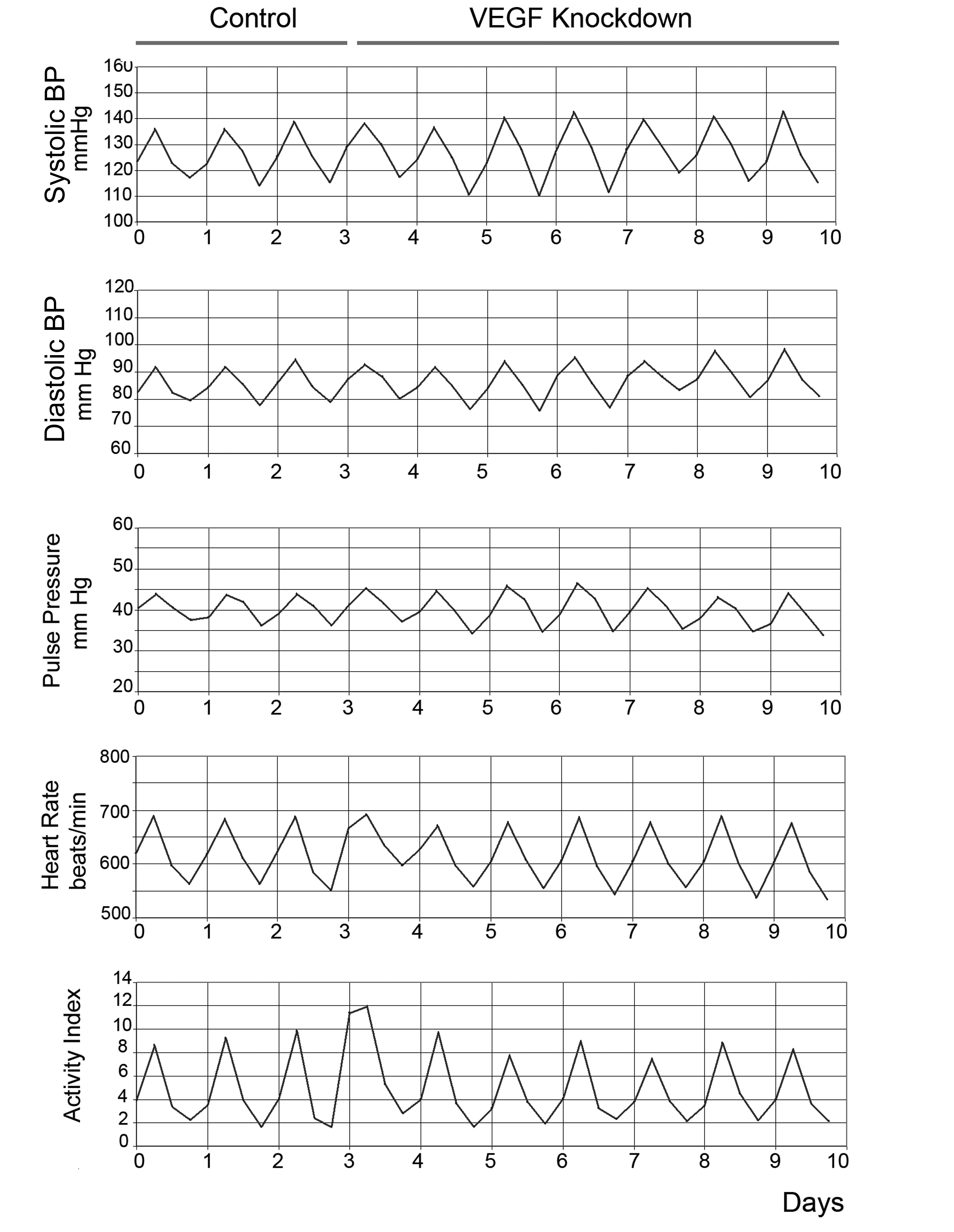

Supplement: Figure S1 — Podocyte VEGF Knockdown mice have normal blood pressure. Graph represents the average over 6 h periods from systolic blood pressure, diastolic blood pressure, pulse pressure and heart rate recorded every 5 min. All parameters show similar patterns during control period (standard diet) and VEGF knockdown period (doxycycline diet), n = 4 mice. (TIF) [file pone.0040589.s001.tif]

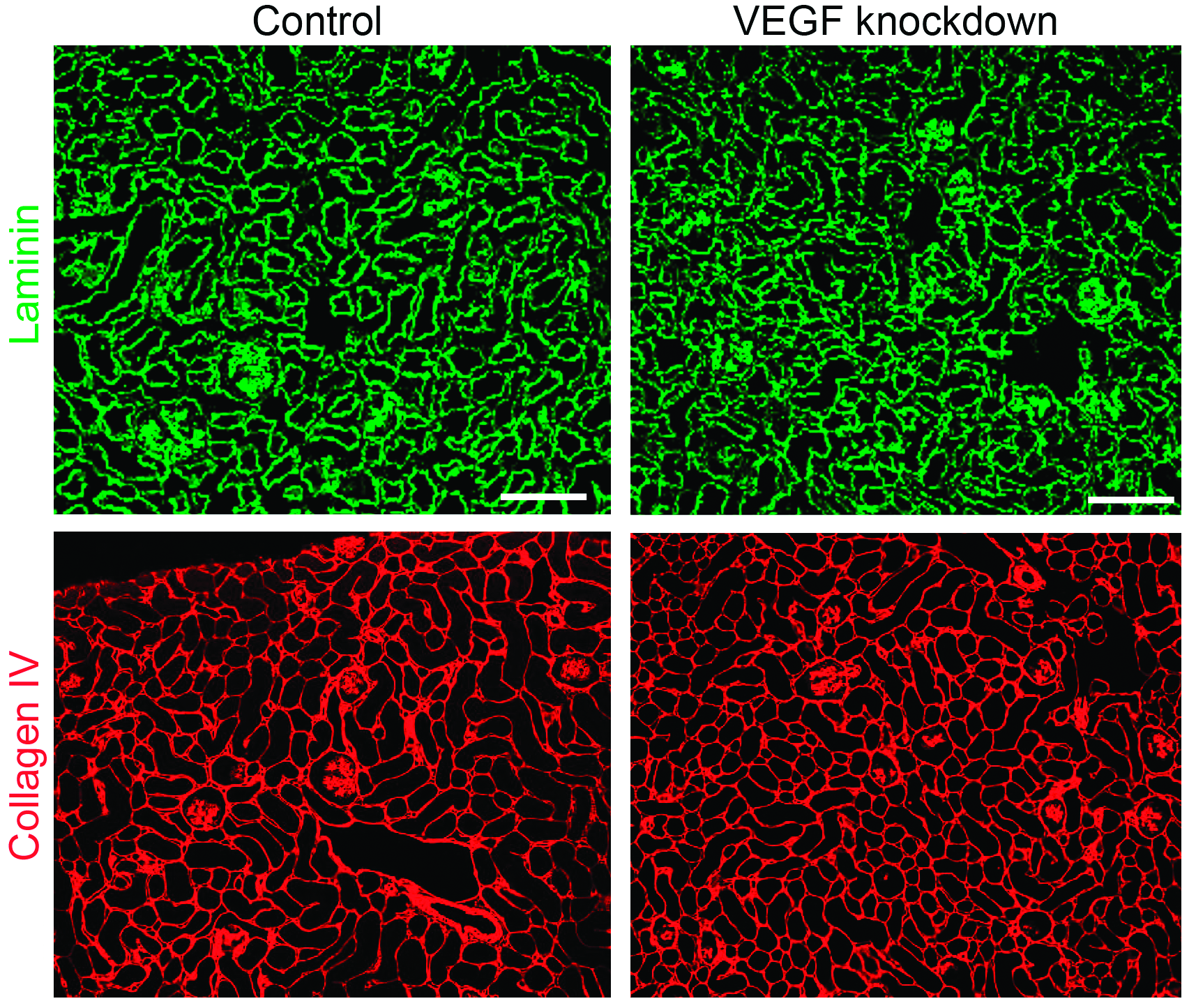

Supplement: Figure S2 — Podocyte VEGF knockdown does not alter total laminin or total collagenIV in the kidney. IHC: laminin (green) and collagenIV (red) low magnification images show similar localization pattern in control and VEGF knockdown kidneys. Scale bars = 100 µm. (TIF) [file pone.0040589.s002.tif]

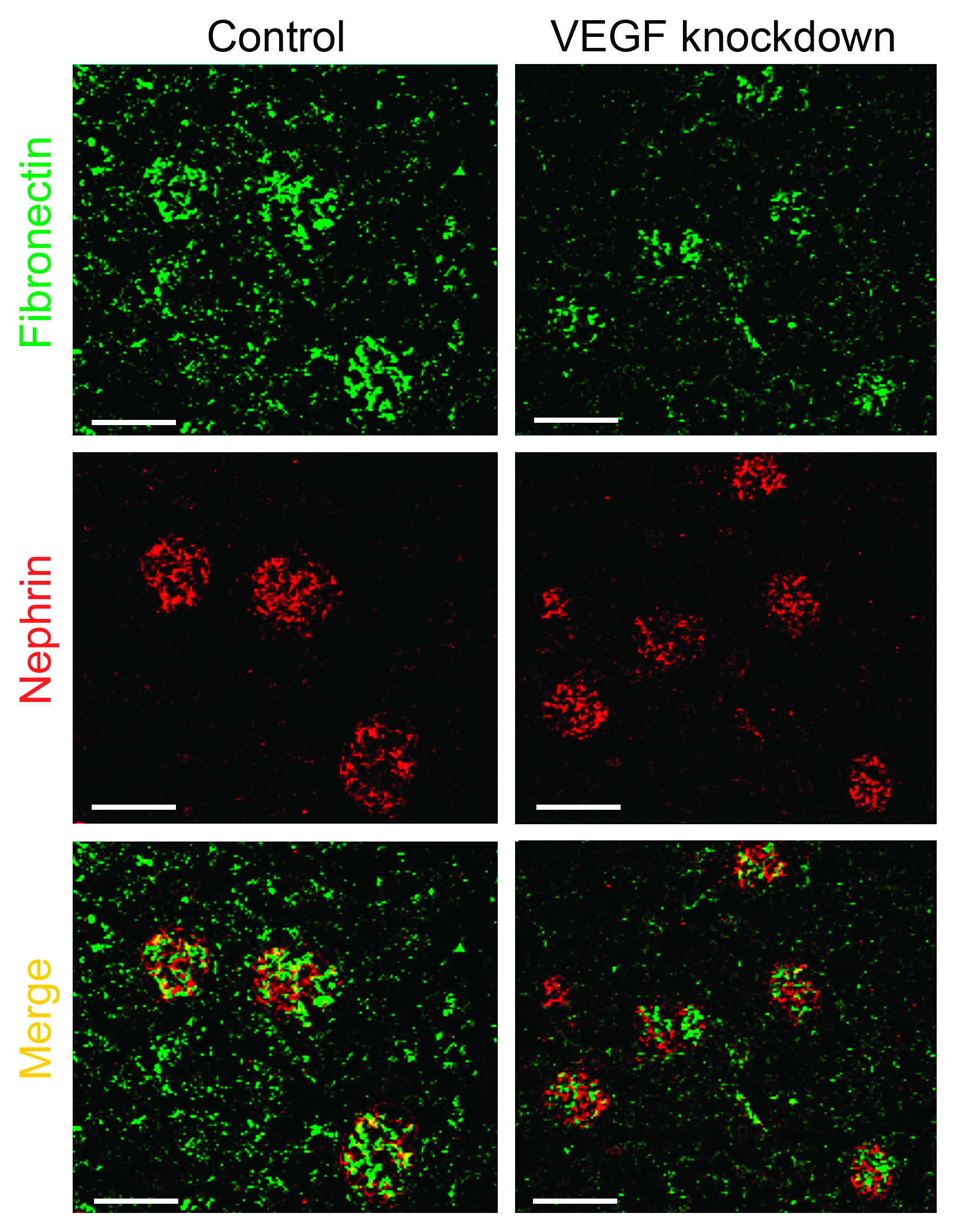

Supplement: Figure S3 — Podocyte VEGF knockdown downregulates glomerular fibronectin. IHC: fibronectin (green) and nephrin (red) low magnification images show decreased fibronectin and preserved nephrin in VEGF knockdown glomeruli. Decreased merged signals (yellow) suggest that fibronectin is decreased in podocytes. Scale bars = 100 µm. (TIF) [file pone.0040589.s003.tif]

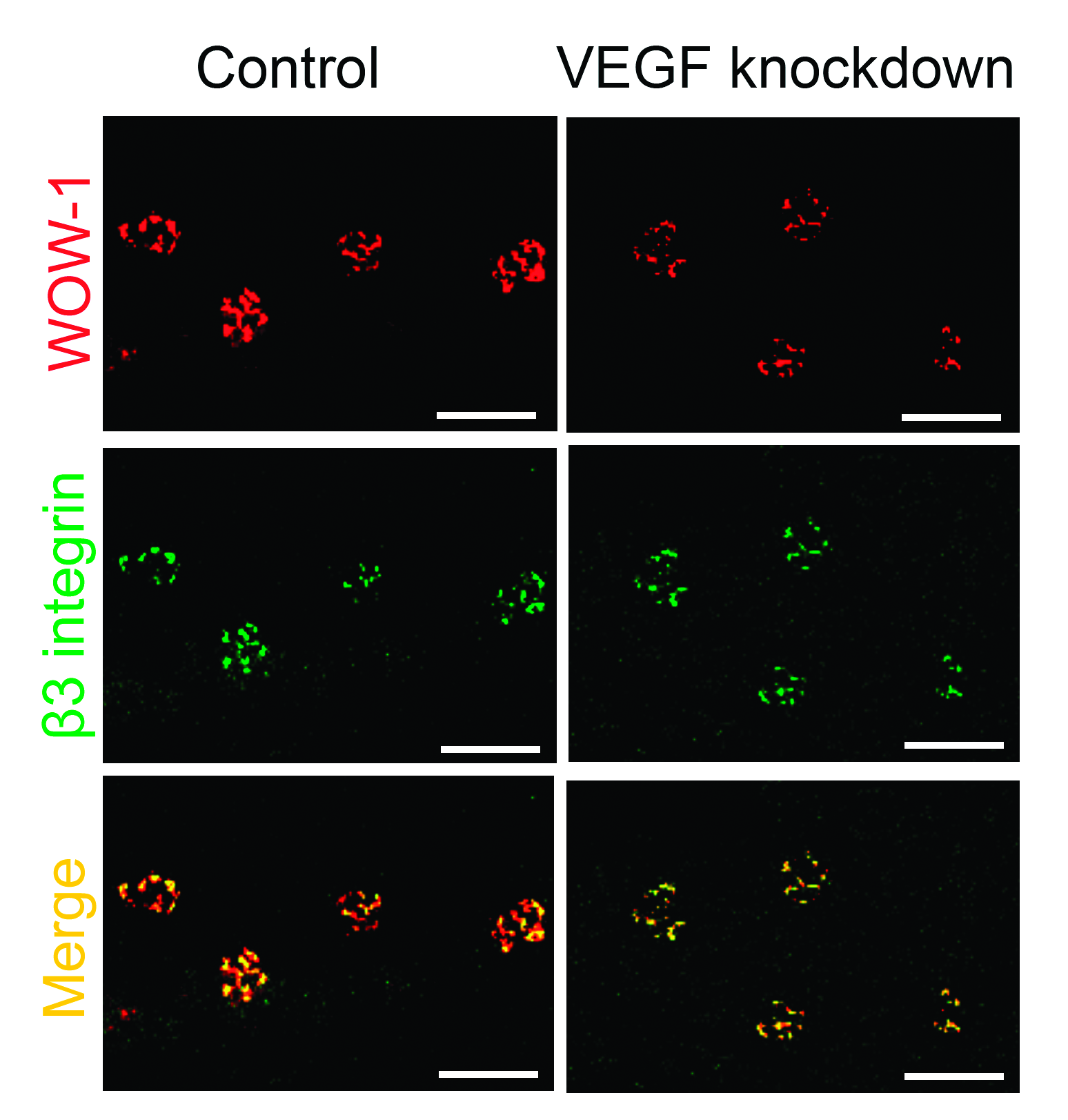

Supplement: Figure S4 — VEGF-A knockdown decreases glomerular alphav beta3 activity. Dual-immunostaining shows decreased active alphavbeta3 integrin (WOW-1) and total beta3 integrin in glomeruli from VEGF knockdown mice, and demonstrates that alphavbeta3 integrin expression is limited to glomeruli; scale bars = 100 µm. (TIF) [file pone.0040589.s004.tif]

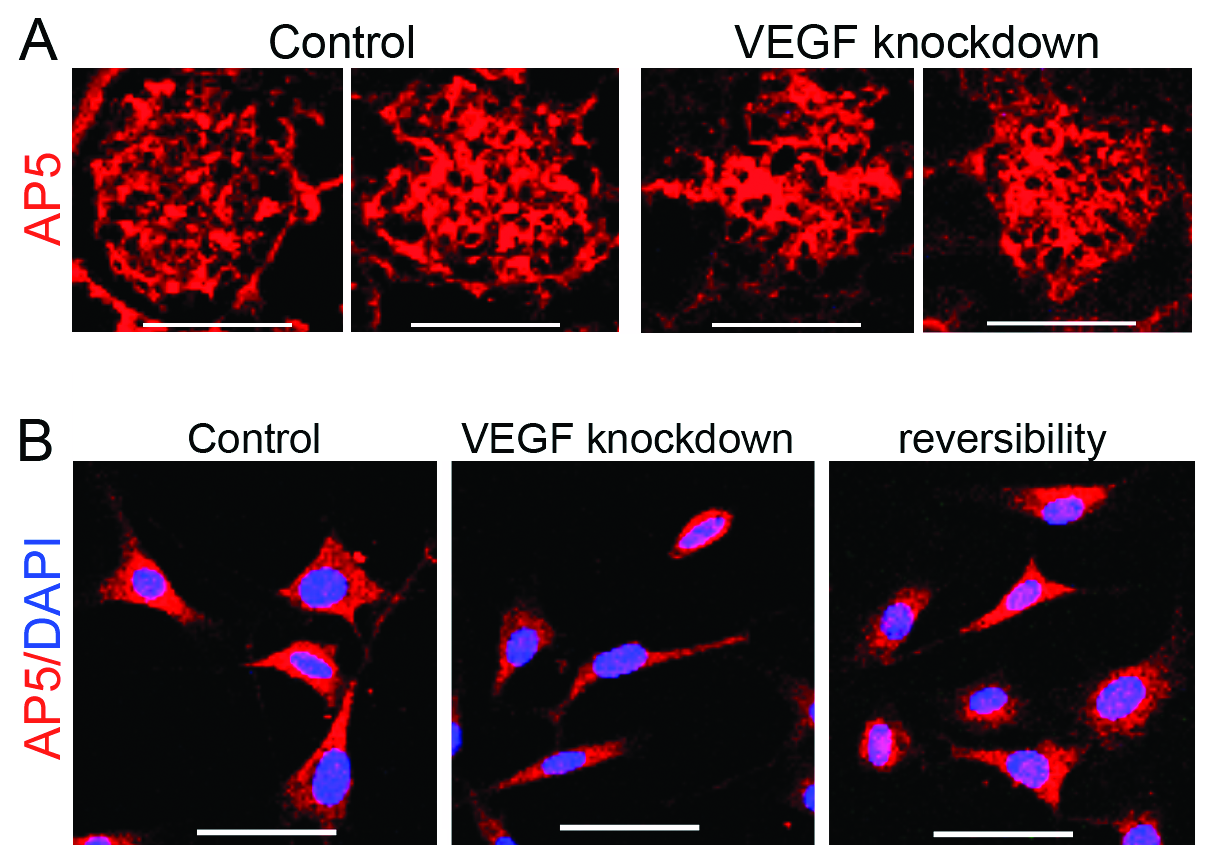

Supplement: Figure S5 — Podocyte VEGF knockdown does not alter outside-in alphav beta3 activation. IHC: AP5 immunolabeling is similar in control and VEGF knockdown glomeruli (A), and podocytes, even after exposure to VEGF165 (B), suggesting that VEGF knockdown does not modulate outside-in alphav beta3 activation. Scale bars = 50 µm. (TIF) [file pone.0040589.s005.tif]

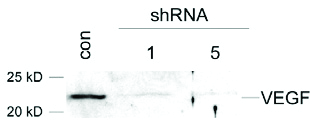

Supplement: Figure S6 — VEGF-A knockdown by shRNA in culture. VEGF shRNA construct was transfected into HeLa-tet-on cells, clones 1 and 5 were induced with 1 µg/ml doxycycline; proteins were extracted after 48 hours and analyzed by western blotting using a polyclonal anti-VEGF antibody (SC#507). Control cells transfected with empty vector are compared with induced clones 1 and 5 showing ∼90% inhibition of protein expression. (TIF) [file pone.0040589.s006.tif]
